# Impact of Genetic Variation in *TLR4* 3′UTR on NSCLC Genetic Susceptibility

**DOI:** 10.1155/2020/7593143

**Published:** 2020-04-09

**Authors:** Hongjiao Wu, Hui Gao, Ang Li, Yuning Xie, Zhenxian Jia, Zhenbang Yang, Hongmei Zhang, Zhi Zhang, Xuemei Zhang

**Affiliations:** ^1^College of Life Science, North China University of Science and Technology, Tangshan 063210, China; ^2^School of Public Health, North China University of Science and Technology, Tangshan 063210, China; ^3^School of Basic Medical Sciences, North China University of Science and Technology, Tangshan 063210, China; ^4^Affliated Tangshan Gongren Hospital, North China University of Science and Technology, Tangshan 063000, China

## Abstract

Toll-like receptors (TLRs) are expressed not only in immune cells but also in a variety of tumor cells. Single-nucleotide polymorphisms (SNPs) located in the TLRs' promoter or the 3′ untranslated region may affect gene expression by affecting the activity of the promoter or regulating the binding of mRNA to miRNA. This study aimed to investigate the association of the SNPs in TLR genes with the susceptibility to NSCLC. This case-control study involved 700 lung cancer patients and 700 healthy controls. All individuals were genotyped for all selected SNPs in TLR genes using polymerase chain reaction (PCR) test-based restriction fragment length polymorphism (PCR-RFLP) and TaqMan SNP genotyping assay. The association of genetic variations in TLRs with the susceptibility to NSCLC was evaluated by unconditional logistic regression with OR (95% CI). After evaluating transcriptional factor or miRNA binding capability by bioinformatics methods, six TLRs were identified for further analysis. We did not find that *TLR3* rs5743303, *TLR4* rs1927914, *TLR4* rs11536891, *TLR5* rs1640816, and *TLR7* rs3853839 were associated with NSCLC risk (*P* > 0.05). Our data showed that *TLR4* rs7869402 C > T polymorphism reduced the risk of NSCLC with OR (95% CI) of 0.63 (0.45–0.89). When stratified by gender and age, the individuals carrying at least one rs7869402T allele significantly decreased the NSCLC risk among males (OR = 0.58, 95% CI = 0.38–0.87) and among youngsters (OR = 0.43, 95% CI = 0.27–0.69). Smoking stratification analysis showed that the rs7869402T allele-containing genotype reduced the risk of NSCLC with OR (95% CI) of 0.50 (0.29–0.87) among smokers but not among nonsmokers (*P* > 0.05). When the individuals were classed by the pathological type, we found that the rs7869402T-containing genotype was associated with the risk of adenocarcinoma (OR = 0.62, 95% CI = 0.41–0.92) but not with that of squamous cell carcinoma (OR = 0.71, 95% CI = 0.44–1.13) and other types (OR = 0.23, 95% CI = 0.03–1.70). Compared with the *TLR4* A_rs1927914_-C_rs7869402_-T_rs11536891_ haplotype, the G_rs1927914_-T_rs7869402_-T_rs11536891_ haplotype was associated with a decreased risk for developing NSCLC with OR (95% CI) of 0.57 (0.41–0.80). These results indicated that the *TLR4* rs7869402 variation affects the genetic susceptibility to NSCLC.

## 1. Introduction

Cancers are major public health problem globally. Worldwide, there are 18.1 million new cancer cases and 9.6 million cancer deaths in 2018, of which lung cancer is the most common one [1, 2]. Non-small-cell lung cancer (NSCLC), as a major type of lung cancer, is the result of interaction of multiple genes and environmental factors, such as smoking, air pollution, and occupational exposure.

In the development of cancer, the fighting of malignant cells with the immune system is an important process. Immune system kills or eliminates malignant transformed cells. In turn, malignant cells evade immune surveillance in order to facilitate their own development. Toll-like receptors are evolutionarily conserved innate immune receptors and belong to the pattern recognition receptor (PRR), which recognizes specific molecular structures of microorganisms (PAMPs and DAMPs). After recognizing PAMPs or DAMPs, TLRs recruit and activate downstream molecules such as TRIF and MyD88 and then activate NF-*κ*B to induce the production of type I interferons and inflammatory factors [3].

TLRs are expressed not only in immune cells but also in a variety of tumor cells. Several studies showed that *TLR2*, *TLR4*, and *TLR9* are overexpressed in lung cancer tissue compared to normal lung tissue [4–6]. The silencing of *TLR4* by siRNA can promote apoptosis and metastasize and inhibit lung cancer cell growth [7, 8]. The elevated *TLR5* expression was prone to improved prognosis among NSCLC patients [9]. The expression of *TLR7* is also associated with the poor prognosis and resistance to neoadjuvant chemotherapy [10].

Single-nucleotide polymorphism (SNP) is widely found in the genome and is the most common type of genetic variation. SNPs located in the promoter region may affect promoter activity by altering the binding capability of the transcription factor. The SNPs located in the 3′ untranslated region may regulate miRNA binding and further affect the efficiency of mRNA translation.

TLRs play an important role in the pathogenesis of various tumors. By bioinformatic analysis, we found six SNPs which may affect the function of *TLR4*. In this study, we explored whether these potential functional variants were associated with the risk of NSCLC.

## 2. Materials and Methods

### 2.1. Study Population

This group-designed case-control study includes 700 NSCLC patients and 700 healthy controls. The NSCLC cases were collected from 2012 to 2014 in Tangshan Gongren Hospital and Renmin Hospital of North China University of Science and Technology in China. All NSCLC cases were histopathologically confirmed. No radiotherapy or antitumor chemotherapy was performed before blood sampling. At the time of sample sampling, the gender, age, pathological type, and the stage of lung cancer patients were not limited. Patients with a previous history of tumor were excluded. Healthy controls were recruited from a physical examination population in Tangshan area during the same period when the cancer patients were involved. All health controls have no history of cancer and frequency match to cases by sex and age (5 years). All participants provided informed consent. The study was supported by the Institutional Review Board of North China University of Science and Technology.

### 2.2. SNP Selecting

Based on the data in the dbSNP database and Ensembl database, we screened the SNPs which were located in the promoter region and 3′ untranslated region (UTR) of TLRs (*TLR3*, *TLR4*, *TLR5*, and *TLR7*). The SNPs with the frequency of minor alleles greater than 0.05 were selected to predict the possible function. For the SNPs in the promoter region of TLRs, transcription factor binding capability was predicted by the online TRANSFAC program. For the SNPs in the 3′ UTR, microRNA binding ability was predicted using the mirSNP and SNPinfo program.

### 2.3. Genotyping of the TLR Variants

**Figure 1:**
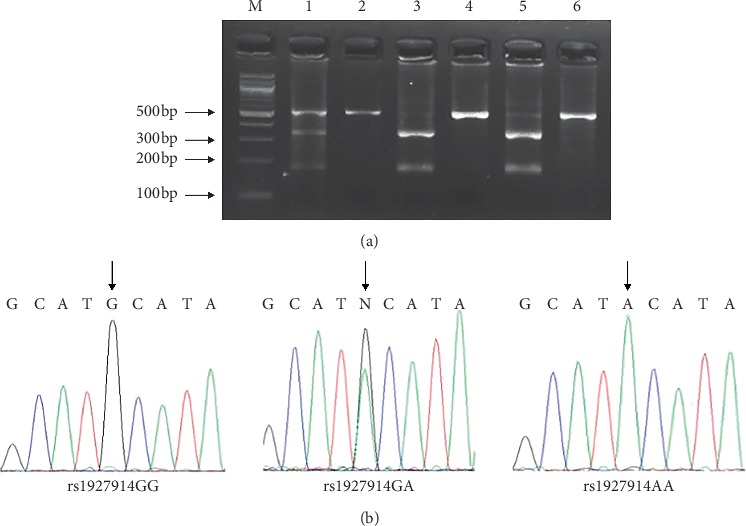
Analysis of TLR4 rs1927914 polymorphism: (a) partial DNA sequence of PCR products with different *TLR4* rs1927914 genotypes; (b) representative gel picture showing PCR-RFLP. M, DNA size markers; lanes 2, 4, and 6, AA genotype; lane 1, GA genotype; lanes 3 and 5, GG genotypes.

**Figure 2:**
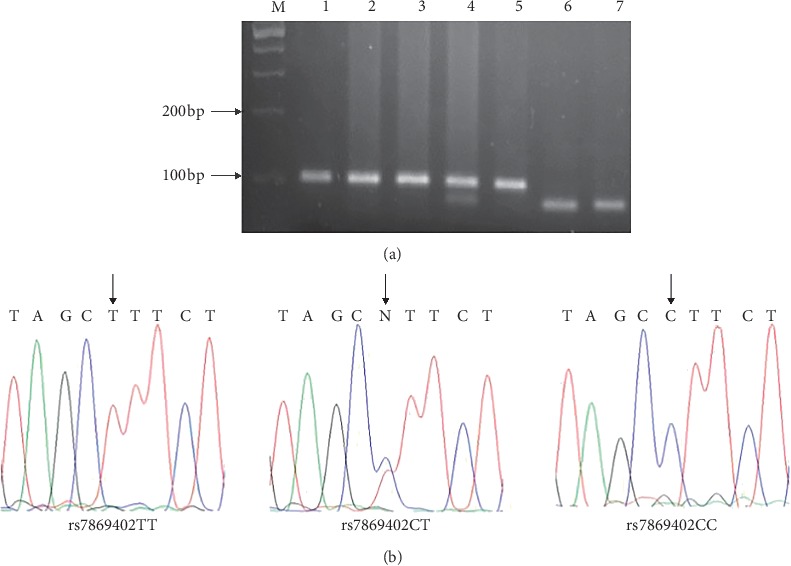
Analysis of TLR4 rs7869402 polymorphism: (a) partial DNA sequence of PCR products with different TLR4 rs7869402 genotypes; (b) representative gel picture showing PCR-RFLP. M, DNA size markers; lanes 1, 2, 3, and 5, CC genotype; lane 4, CT genotype; lanes 6 and 7, TT genotypes.

Polymerase chain reaction (PCR)-restriction fragment length polymorphism (RFLP) analysis and TaqMan SNP genotyping assay were applied for genotyping [11, 12]. Genotyping for *TLR4* rs1927914 and rs7869402 variants was performed by PCR-RFLP. The primer pairs used to identify *TLR4* rs1927914 or *TLR4* rs7869402 polymorphisms were 5′- TAGCATGAGAAATGAGGAAGTAAGGG-3′/5′- GAGCTATGATGAGGATTGAAAATGTGG-3′ and 5′- TGGGATCCCTCCCCTGTAGC-3′/5′-AGGAGCATTGCCCAACAGG-3′. PCR was performed in 6 *μ*L PCR reaction mixture with 2 × Taq PCR StarMix, 0.1 *μ*M each primer, and 20 ng genomic DNA. The thermal cycling conditions for *TLR4* rs1927914 and rs7869402 variants were 5 min at 94°C followed by 30 cycles (30°s at 94°C, 30°s at 58°C, and 30°s at 72°C) and 5 min at 72°C. PCR amplification products for *TLR4* rs1927914 A > G (524 bp) and *TLR4* rs7869402 C > T (102 bp) were digested by Nsi *I* (NEB, Ipswich, MA, USA) and Alu *I* (NEB, Ipswich, MA, USA). DNA sequencing was used to confirm the accuracy of PCR-RFLP results (Figures 1 and 2). A 10% sample was randomly selected and regenotyped, and all results were found to be 100% consistent. Genotyping for other genetic variants (*TLR3* rs5743303, *TLR5* rs1640816, and *TLR7* rs3853839) was performed using TaqMan SNP genotyping assays (C_27310258, C_8812434, and C_2259573) (Thermo Fisher Scientific, Waltham, USA).

### 2.4. Statistical Analysis

All analyses were conducted with SPSS23.0 (SPSS Inc., Chicago, USA). The basic information (sex, age group, and smoking status) of the subjects was analyzed by *χ*^*2*^ test. Hardy–Weinberg equilibrium (HWE) of all tested SNPs among controls was estimated by *χ*^*2*^ test. After adjusting for possible confounding factors, the association of genetic variations in *TLRs* with the susceptibility to NSCLC was evaluated by unconditional logistic regression with OR (95% CI).

## 3. Results

### 3.1. Subject Characteristics

The basic information of 700 NSCLC patients and 700 health controls is summarized in Table 1. The distributions of gender and age in the case group are consistent with those in the group of controls (*P* > 0.05). The proportion of smokers in the case group was 44.4%, which was higher than that among health controls (28.1%) (*P* < 0.01). The distribution of cumulative smoking between the case and control groups has no significant difference (*P*=0.773). Among all NSCLC cases, there are 402 patients with adenocarcinomas (57.4%), 279 patients with squamous cell carcinomas (38.6%), and 28 patients with other pathological types (15 adenosquamous carcinoma, 5 large-cell cancer, and 8 cases of bronchoalveolar carcinoma).

### 3.2. Association of TLR Variants with the Risk of NSCLC

The SNP information is shown in Table 2. The relationship between each genetic variant and the susceptibility to NSCLC is shown in Table 3. The frequency of *TLR4* rs7869402 CC, CT, and TT genotypes in the case group and controls was 90.7%, 9.0%, and 0.3% and 86.0%, 13.3%, and 0.7%. The results showed that individuals with at least one T allele had reduced the risk of NSCLC compared with CC genotype carriers (OR = 0.63, 95% CI = 0.45–0.89). We did not find any statistical difference in the distribution of other SNPs in *TLR3, TRL4*, and *TLR7* between cases and controls.

### 3.3. Stratification Analysis of the *TLR4* Variants and NSCLC Risk

To further analyze the relationship between *TLR4* rs7869402 genetic variation and NSCLC risk, we performed a stratified analysis by gender, age, smoking status, and pathological type (Table 4). When stratified by gender, males with at least one T allele had a lower risk of NSCLC than those with the CC genotype with OR (95% CI) of 0.58 (0.38–0.87). *TLR4* rs7869402 was not associated with NSCLC risk among females with OR (95% CI) of 0.78 (0.43–1.41). In stratified analysis by age, the younger subjects (age ≤ 60) carrying at least one T allele had a decreased risk of NSCLC with OR (95% CI) of 0.43 (0.27–0.69), which was not found among old subjects. When stratified by smoking status, compared with CC genotype carriers, the T allele-containing genotype contributed to a reduced risk of NSCLC (OR = 0.50, 95% CI = 0.29–0.87) among smokers but among nonsmokers (OR = 0.74, 95% CI = 0.49–1.13). Further stratification analysis of smoking levels showed that the T allele-containing genotype contributed to decreased risk of NSCLC among light smokers (≤30 packs/year) with OR (95% CI) of 0.46 (0.23–0.92) but not among heavy smokers with OR (95% CI) of 0.44 (0.17–1.14). A stratified analysis of different pathological types revealed that individuals with at least one T allele were associated with the risk of adenocarcinoma with OR (95% CI) of 0.62 (0.41–0.92) but not with the risk of squamous cell carcinoma with OR (95% CI) of 0.71 (0.44–1.13) and other types of NSCLC with OR (95% CI) of 0.23 (0.03–1.70).

### 3.4. Haplotype Analysis of *TLR4* Variants

In order to evaluate the impact of the interaction of multiple SNPs on the risk of NSCLC, we tested the association of statistically inferred haplotypes with the risk of NSCLC using SHE-sis online program. Our results showed that the distribution of the *TLR4* haplotype of G_rs1927914_-T_rs7869402_-T_rs11536891_ was statistically different between NSCLC patients and health controls (Table 5). Compared with the *TLR4* A_rs1927914_-C_rs7869402_-T_rs11536891_ haplotype, the G_rs1927914_-T_rs7869402_-T_rs11536891_ haplotype was associated with a decreased risk for developing NSCLC with OR (95% CI) of 0.57 (0.41–0.80).

## 4. Discussion

This study explored the relationship between genetic variation in TLR genes and the susceptibility to NSCLC. We screened 6 SNPs in the promoter and 3′UTR of TLRs that may affect the gene expression by bioinformatics prediction. Our finding showed that *TLR4* rs7869402 C > T variation decreased the risk of NSCLC. However, *TLR3* rs5743303, *TLR4* rs1927914, *TLR4* rs11536891, *TLR5* rs1640816, and *TLR7* rs3853839 genetic variants were not associated with NSCLC risk. These studies suggest that *TLR4* rs7869402 C > T variation may be involved in the pathogenesis and progression of NSCLC.

Human TLR family included a total of 10 gene subtypes [13]. *TLR4* is one of the earliest and most widely studied toll-like receptors. *TLR4* is located on chromosome 9 and contains four exons. *TLR4* involved in tumor occurrence and development by inducing M2 macrophage infiltration and angiogenesis in tumor microenvironment and participating in the process of apoptosis, MyD88-dependent and independent signal transduction, or other biological processes [14–16]. Studies have shown that *TLR4* is overexpressed in lung cancer tissues, and the knockdown of *TLR4* can promote apoptosis of A549 cells and inhibit the growth of tumor cells [17]. Therefore, *TLR4* may become a susceptibility biomarker for early screening of lung cancer and improve the survival rate of lung cancer patients. In this study, we first demonstrated the relationship between the key variants in the regulation region of TLRs and the risk of NSCLC and found that the *TLR4* rs7869402 C > T was associated with the risk of NSCLC. In an ovarian cancer study, researchers found that *TLR4* rs7869402 variation reduced the overall survival of ovarian cancer [18]. In an oral squamous cell carcinoma research, *TLR4* rs7869402 polymorphism had no association with cancer development and progression-free survival [19]. For other SNPs of TLR4, rs1927914 affects the risk of various tumors, including liver cancer [20], prostate cancer [21], gastric cancer [22], and malignant melanoma [23] but not the risk of hepatocellular carcinoma [24]. Shi et al. found no correlation between *TLR4* rs11536891 mutation and prostate cancer risk or mortality, while Tsilidis KK et al. showed that *TLR4* rs11536891 was associated with susceptibility to colorectal cancer [25].

The development of tumors is related to age and gender. In the gender and age stratification analysis, we found that at least one *TLR4* rs7869402T allele in the male and low-age groups had a reduced risk of lung cancer. In addition, environmental factors such as smoking, air pollution, and occupational environment are considered to be risk factors for lung cancer [26]. *TLR4* directly interacts with the environment, which may have a contributing effect on lung cancer. Smoking stratification analysis showed that *TLR4* rs7869402T allele reduced the risk of NSCLC among smokers but not among nonsmokers. These results suggest that *TLR4* rs7869402 genetic variation may affect the risk of lung cancer due to genetic and environmental interactions.

The key SNPs in the regulation region of TLRs may alter the function of genes, which in turn effects on the progress of lung cancer. In conclusion, our results provided new evidence that *TLR4* contributed to the progress of lung cancer.

## Figures and Tables

**Table 1:** Distributions of selected characteristics of NSCLC cases and control subjects.

Variables	Cases (*n* = 700)		Controls (*n* = 700)		*P* value^a^
	No	(%)	No	(%)	
Gender					0.821
Male	465	66.4	469	67.0	
Female	235	33.6	231	33.0	
Age					1.000
≤50	125	17.9	124	17.7	
51–60	261	37.3	262	37.4	
61–70	225	32.1	224	32.0	
>70	89	12.7	90	12.9	
Smoking status					0.000
Nonsmoker	389	55.6	503	71.9	
Smoker	311	44.4	197	28.1	
Pack year of smoking					0.773
≤30	184	59.2	114	57.9	
>30	127	40.8	83	42.1	
Histological types					
Adenocarcinoma	402	57.4			
Squamous cell	270	38.6			
Other carcinomas	28	4.0			

^a^Two-sided *χ*^2^ test.

**Table 2:** General information of SNPs and Hardy–Weinberg test.

Gene	Position	SNP	Region	Allele gene	MAF	*P* value
TLR3	chr4:186067699	rs5743303	5′UTR	A/T	0.171	0.928
TLR4	chr9:117702447	rs1927914	5′UTR	G/A	0.490	0.522
TLR4	chr9:117715754	rs7869402	3′UTR	C/T	0.109	0.502
TLR4	chr9:117717059	rs11536891	3′UTR	T/C	0.143	0.391
TLR5	chr1:223145246	rs1640816	5′UTR	G/A	0.087	0.444
TLR7	chrX:12889539	rs3853839	3′UTR	C/G	0.402	0.790

**Table 3:** Genotype frequencies of SNPs in TLR genes and their association with NSCLC.

Genotypes	Cases (*n* = 700)		Controls (*n* = 700)		OR (95% CI)^a^	*P* value
	No	(%)	No	(%)		
TLR3 rs5743303						
AA	514	73.4	508	72.6		
AT	163	23.3	177	25.3	0.89 (0.69–1.15)	0.366
TT	23	3.3	15	2.1	1.46 (0.75–2.86)	0.270
TLR4 rs1927914						
AA	225	32.1	233	33.3		
AG	351	50.1	346	49.4	0.96 (0.75–1.22)	0.708
GG	124	17.8	121	17.3	0.97 (0.76–1.33)	0.841
TLR4 rs7869402						
CC	635	90.7	602	86.0		
CT	63	9.0	93	13.3	0.65 (0.46–0.91)	0.013^b^
TT	2	0.3	5	0.7	0.38 (0.07–2.01)	0.254
CT + TT	65	9.3	98	14.0	0.63 (0.45–0.89)	0.008^b^
TLR4 rs11536891						
TT	586	83.7	605	86.4		
CT	112	16.0	93	13.3	1.22 (0.90–1.65)	0.203
CC	2	0.3	2	0.3	1.19 (0.90–1.65)	0.862
CT + CC	114	16.3	95	13.6	1.22 (0.90–1.64)	0.200
TLR5 rs1640816						
GG	573	81.9	567	81.0		
AG	117	16.7	128	18.3	0.85 (0.64–1.23)	0.263
AA	10	1.4	5	0.7	0.97 (0.71–1.33)	0.841
TLR7 rs3853839						
Male						
G	357	51.0	352	50.3		
C	108	15.4	117	16.7	0.94 (0.69–1.28)	0.690
Female						
GG	136	19.4	138	19.7		
CG	83	11.9	82	11.7	1.01 (0.69–1.49)	0.960
CC	16	2.3	11	1.6	1.40 (0.62–3.15)	0.415

^a^Adjusted for age, gender, and smoking status.

**Table 4:** The association of the *TLR4* rs7869402 variant with NSCLC risk by selected variables.

Variables	Genotypes (cases/controls)			TT + CT/CC model OR (95% CI)^a^	*P* value (TT + CT/CC)	CT/CC model OR (95% CI)^a^	*P* value (CT/TT)
	CC	CT	TT				
Gender							
Male	423/399	41/67	1/3	0.58 (0.38–0.87)	0.009	0.58 (0.38–0.89)	0.014
Female	212/203	22/26	1/2	0.78 (0.43–1.41)	0.414	0.65 (0.46–0.91)	0.478
Age							
≤60	357/328	28/54	1/4	0.43 (0.27–0.69)	0.001	0.44 (0.27–0.72)	0.001
>60	278/274	35/39	1/1	0.96 (0.59–1.57)	0.868	0.96 (0.59–1.59)	0.881
Smoking status							
Nonsmoker	350/436	38/63	1/4	0.74 (0.49–1.13)	0.168	0.77 (0.50–1.19)	0.241
Smoker	285/166	25/30	1/1	0.50 (0.29–0.87)	0.013	0.49 (0.28–0.87)	0.014
Pack year of smoking							
≤30	167/94	16/20	1/0	0.46 (0.23–0.92)	0.029	0.42 (0.20–0.86)	0.018
>30	118/72	91/10	0/1	0.44 (0.17–1.14)	0.091	0.48 (0.18–1.27)	0.137
Histological types							
Adenocarcinoma	365/602	35/93	2/5	0.62 (0.41–0.92)	0.019	0.61 (0.41–0.93)	0.021
Squamous cell	243/602	27/93	0/5	0.71 (0.44–1.13)	0.145	0.74 (0.46–1.19)	0.214
Other carcinomas	27/602	1/93	0/5	0.23 (0.03–1.70)	0.149	0.24 (0.03–1.79)	0.164

^a^Data were calculated by unconditional logistic regression and adjusted for age, gender, and smoking status, where they were appropriate.

**Table 5:** Haplotype frequencies of *TLR4* among cases and controls and their association with NSCLC.

Haplotype	Cases (2*n* = 1400) 2*n* (%)	Controls (2*n* = 1400) 2*n* (%)	OR (95% CI)	*P* value
A_rs1927914_-C_rs7869402_-T_rs11536891_	788 (56.3)	787 (56.2)	1.00	
G_rs1927914_-C_rs7869402_-C_rs11536891_	99 (7.1)	86 (6.1)	1.18 (0.87–1.59)	0.283
G_rs1927914_-C_rs7869402_-T_rs11536891_	430 (30.7)	414 (29.6)	1.07 (0.91–1.26)	0.417
G_rs1927914_-T_rs7869402_-T_rs11536891_	59 (4.2)	100 (7.1)	0.57 (0.41–0.80)	0.001
A_rs1927914_-C_rs7869402_-C_rs11536891_	16 (1.1)	11 (0.8)	NC	NC
A_rs1927914_-T_rs7869402_-T_rs11536891_	7 (0.5)	3 (0.2)	NC	NC
G_rs1927914_-T_rs7869402_-C_rs11536891_	0 (0.0)	0 (0.0)	NC	NC
A_rs1927914_-T_rs7869402_-C_rs11536891_	1 (0.1)	0 (0.0)	NC	NC

NC, not calculated.

## Data Availability

The data used to support the findings of this study are included within the article.
